# NMDAR Neurotransmission Needed for Persistent Neuronal Firing: Potential Roles in Mental Disorders

**DOI:** 10.3389/fpsyt.2021.654322

**Published:** 2021-04-09

**Authors:** Shengtao Yang, Hyojung Seo, Min Wang, Amy F. T. Arnsten

**Affiliations:** ^1^Department of Neuroscience, Yale University School of Medicine, New Haven, CT, United States; ^2^Department of Psychiatry, Yale University School of Medicine, New Haven, CT, United States

**Keywords:** NMDAR (NMDA receptor), prefrontal cortex, cingulate cortex, working memory, depression

## Abstract

The dorsolateral prefrontal cortex (dlPFC) generates the mental representations that are the foundation of abstract thought, and provides top-down regulation of emotion through projections to the medial PFC and cingulate cortices. Physiological recordings from dlPFC Delay cells have shown that the generation of mental representations during working memory relies on NMDAR neurotransmission, with surprisingly little contribution from AMPAR. Systemic administration of low “antidepressant” doses of the NMDAR antagonist, ketamine, erodes these representations and reduces dlPFC Delay cell firing. In contrast to the dlPFC, V1 neuronal firing to visual stimuli depends on AMPAR, with much less contribution from NMDAR. Similarly, neurons in the dlPFC that respond to sensory events (cue cells, response feedback cells) rely on AMPAR, and systemic ketamine increases their firing. Insults to NMDAR transmission, and the impaired ability for dlPFC to generate mental representations, may contribute to cognitive deficits in schizophrenia, e.g., from genetic insults that weaken NMDAR transmission, or from blockade of NMDAR by kynurenic acid. Elevated levels of kynurenic acid in dlPFC may also contribute to cognitive deficits in other disorders with pronounced neuroinflammation (e.g., Alzheimer's disease), or peripheral infections where kynurenine can enter brain (e.g., delirium from sepsis, “brain fog” in COVID19). Much less is known about NMDAR actions in the primate cingulate cortices. However, NMDAR neurotransmission appears to process the affective and visceral responses to pain and other aversive experiences mediated by the cingulate cortices, which may contribute to sustained alterations in mood state. We hypothesize that the very rapid, antidepressant effects of intranasal ketamine may involve the disruption of NMDAR-generated aversive mood states by the anterior and subgenual cingulate cortices, providing a “foot in the door” to allow the subsequent return of top-down regulation by higher PFC areas. Thus, the detrimental vs. therapeutic effects of NMDAR blockade may be circuit dependent.

## Introduction

The recent discovery that the NMDA receptor (NMDAR) antagonist, ketamine, can produce rapid, antidepressant actions has stirred interest in the possible mechanisms underlying these therapeutic effects, and why blockade of NMDAR can produce such a swift change in mood. The current review discusses how NMDAR-calcium mechanisms are needed for sustained neural representations, e.g., such as the persistent representation of visual space in working memory by circuits in the dorsolateral prefrontal cortex (dlPFC), and suggests that parallel mechanisms in the cingulate circuits mediating mood and emotion may be overactivated in depression, and aided by NMDAR blockade (1, 2).

NMDAR are heterotetramers composed of GluN1 and GluN2 (A-D) or GluN3 (A-B) subunits- usually with two GluN1 and two GluN2 subunits (3). The GluN2B subunit, also known as the NR2B subunit, has been of particular interest, as it closes more slowly than the common, GluN2A subunit, and fluxes high levels of calcium into the neuron (4). Although previous research in rodent classic circuits had found that NMDA-GluN2B were mostly at extra-synaptic locations (5), or played a role only in immature neurons (6), more recent research has shown that GluN2B play a critical, synaptic role in the primate cortical circuits mediating higher cognition, providing the synaptic events that generate sustained representations of visual space in working memory in the dlPFC (7, 8). The high levels of calcium influx into spines may be especially important for maintaining a depolarized post-synaptic membrane, permitting continued neural firing needed to sustain representations over long time periods (9). Recent research has also shown that expression of NMDAR with GluN2B subunits encoded by the *GRIN2B* gene expands across primate cortical evolution (10), and across the cortical hierarchy in humans, with especially high levels in association and limbic cortices such as the anterior cingulate cortex (11). The following paper explores the hypothesis that the critical role of GluN2B in generating sustained representations in dlPFC may extend to the generation of aversive mood state by the anterior and subgenual cingulate cortices, and that NMDAR blockade by ketamine may be helpful by relieving this self-perpetuating, aversive network activity.

The paper will briefly review PFC circuits in primates and their regulation of the cingulate cortices, and then discuss the critical role of NMDAR for generating mental representations in dlPFC, the expansion in NMDAR-GluN2B transmission across the cortical hierarchy and across cortical evolution, and the role of NMDAR-GluN2B in the cingulate cortices mediating affective pain responses and depression. It will briefly discuss how stress exposure impairs higher PFC regulation, and will close with an exploration of the idea that ketamine's rapid antidepressant actions may involve blocking mental representations of aversive mood state in the cingulate cortices.

## Primate Prefrontal Cortical Circuits

**Figure 1:**
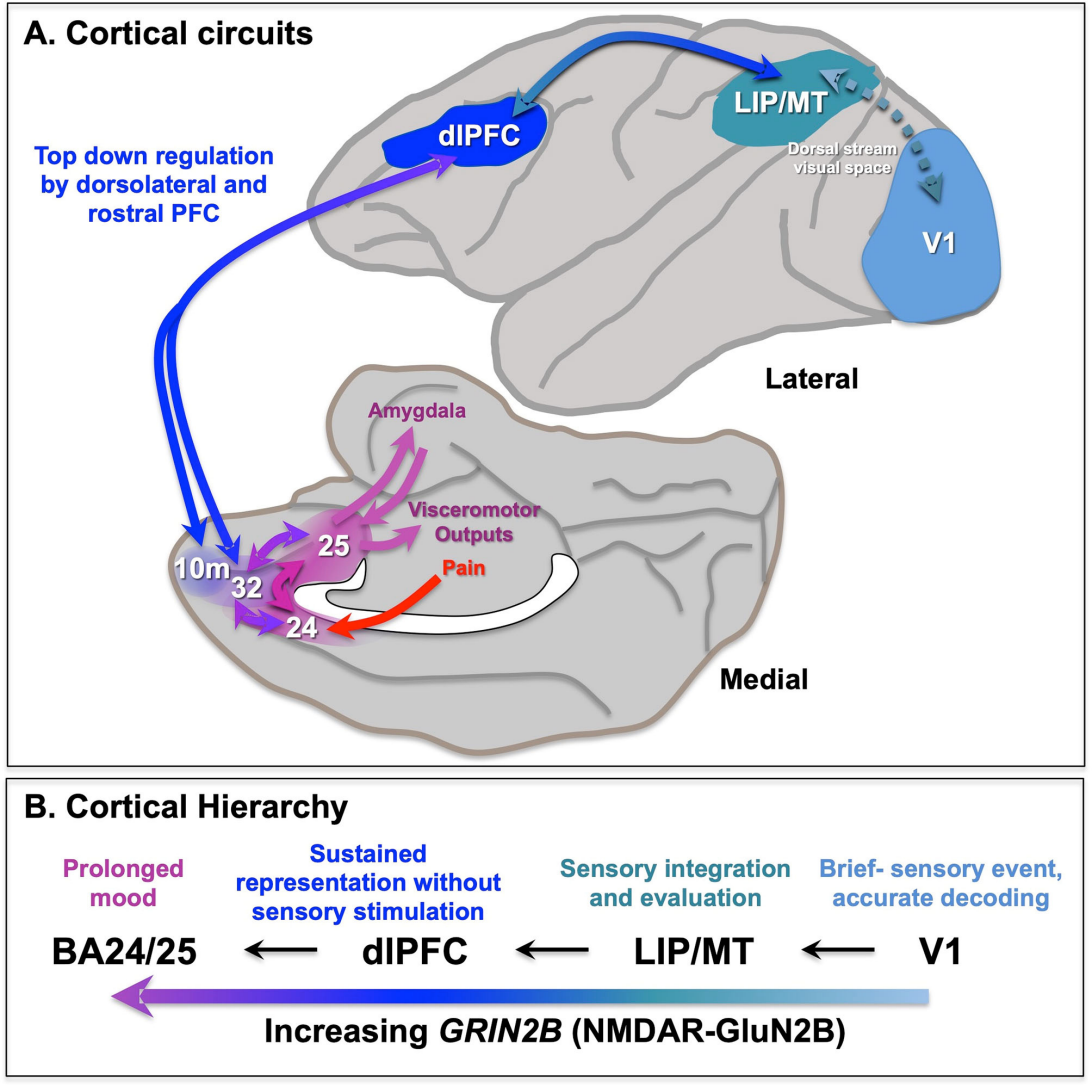
Primate cortical circuits. **(A)** Schematic diagram of circuits in the rhesus monkey cortex, where the lateral surface represents the outer world, and the medial and orbital surface represents inner state. The dorsal stream is shown on the lateral surface, where dlPFC represents visual space in working memory, and generates the goals for top-down regulation of emotion. The medial surface shows the pathways mediating the emotional response to pain, arising from medial thalamic projections to the insular cortex (not shown) and the anterior cingulate cortex BA24, which both project to BA25 (subgenual cingulate). BA25 is a major center for visceromotor outputs, e.g., to the amygdala, brainstem, and hypothalamus to alter heart rate. These cingulate cortices are often overactive in depression, and a target of DBS treatments. The dlPFC provides top-down regulation of emotion through indirect projections to BA25 via areas BA10m and BA32, and direct projections to BA24 (not shown). **(B)** The increasing timescales across the primate cortical hierarchy, and their relationship to GRIN2B expression. Based on (11) and (9). LIP, lateral intraparietal cortex; MT, middle temporal visual cortex.

The PFC greatly expands and differentiates over brain evolution, allowing representations of information in the absence of sensory stimulation. The primate PFC is topographically organized across multiple dimensions, e.g., with “simpler” representative functions found more caudally and more complex (e.g., metacognition) more rostrally in the frontal pole (12, 13). There are also topographic differences across the dorsolateral to ventromedial dimensions (14), where the dlPFC represents the outer world (e.g., with inputs from parietal areas that process visual space, Figure 1A), while the ventral and medial PFC regions represent the inner world, including taste and olfaction combining to represent flavor in orbital (ventral) PFC, and projections from the medial thalamus to the medial anterior cingulate cortex (ACC, BA24) mediating the emotional aspects of pain (Figure 1A). Neurons in the dorsomedial PFC also can represent persistent signatures of loss during a competitive game (15), and anterior cingulate neurons respond to errors (16), suggesting these regions are also activated by aversive psychological events. This information is relayed to the subgenual cingulate (BA25) that has extensive visceromotor connections to induce the physical aspects of the emotional response to pain [Figure 1A; (14)]. For example, BA25 projects to the amygdala, and the hypothalamus and brainstem to effect the autonomic nervous system and facial expression, and to the periaqueductal gray and medial subthalamic nucleus to alter behavioral response (14, 17–19), e.g., “freezing” behavior in response to a threat.

The more newly evolved, rostral and lateral areas of PFC provide top-down regulation of the more primitive medial and caudal areas. For example, the dlPFC can regulates emotion via direct projections to BA24 (20, 21), and indirect projections to BA25 via BA10m or BA32 to BA25 (22, 23) (Figure 1A). The pathways from dlPFC to BA32 and then to BA25 are now known in great detail at the ultrastructural level (23–25), showing how dlPFC and BA32 are positioned to either inhibit or activate emotional responses by BA25.

An important note about species differences: rodents do not have rostral PFC areas (e.g., frontal pole) or a dlPFC, and even the medial and orbital PFC areas they do have are much less developed and differentiated than those in primates (26). Indeed, the dorsal to ventral topography of medial PFC subregions appears to be reversed from rodent to monkey, with the most ventral BA25 activating the stress response in monkeys, but inhibiting it in rodents (27). This may be due to the medial PFC being less differentiated in rodents, with a dorsal-ventral gradient in many medial PFC connections (28). Thus, the actual circuit connections, e.g., with excitatory vs. inhibitory neurons in amygdala, need to be identified for proper interpretation.

## The Critical Role of NMDAR-GluN2B in the Generation of Mental Representations by the dlPFC

**Figure 2:**
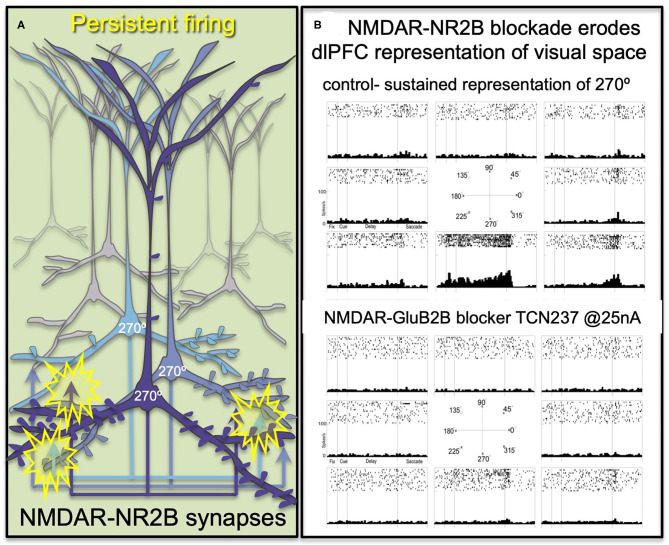
The persistent firing of dlPFC Delay cells depends on NMDAR with GluN2B subunits. **(A)** Schematic illustration of the recurrent excitatory microcircuits in deep layer III of dlPFC that generate persistent firing. **(B)** A dlPFC Delay cell that represents the spatial position of 270° during a spatial working memory task, maintaining firing across the delay period for only that preferred location. Iontophoresis of the selective NMDAR- GluN2B antagonist, TCN237, completely blocks the ability of the neuron to generate representations of visual space.

The primate dlPFC has the remarkable ability to generate and sustain mental representations without sensory stimulation, the foundation of abstract thought (29). dlPFC “Delay cells” are able to maintain persistent firing across the delay period in a working memory task, sustaining representations over many seconds e.g., remembering a position in visual space (30). “Delay cells” appear to reside in pyramidal cell microcircuits in deep layer III of the dlPFC that have extensive recurrent excitatory connections [Figure 2A; (29, 31)], as well as lateral inhibition from parvalbumin-containing interneurons to refine spatial tuning (29, 32). The persistent firing of Delay cells across the delay period depends on NMDAR stimulation (7), a finding predicted by computational models (33). Thus, iontophoresis (local electrical application) of low doses of NMDAR antagonists, including antagonists that selectively block those with GluN2A or GluN2B subunits, markedly reduces Delay cell firing (7). An example is shown in Figure 2B, where under control conditions a Delay cell can sustain the representation of the cue that had been flashed at 270° over many seconds in working memory. However, the Delay cell is no longer able to represent spatial information in working memory following the local iontophoretic blockade of NMDAR GluN2B with the antagonist, TCN237.

Immunoelectron microscopy (immunoEM) showed that NMDAR-GluN2B are expressed exclusively within the post-synaptic density (PSD) in layer III dlPFC spines, and are *not* extra-synaptic, consistent with their direct mediation of neurotransmission (7). The ability of GluN2B subunits to flux large amounts of calcium may be a key aspect of why they support persistent firing in computational models (33) and in Delay cells (7).

**Figure 3:**
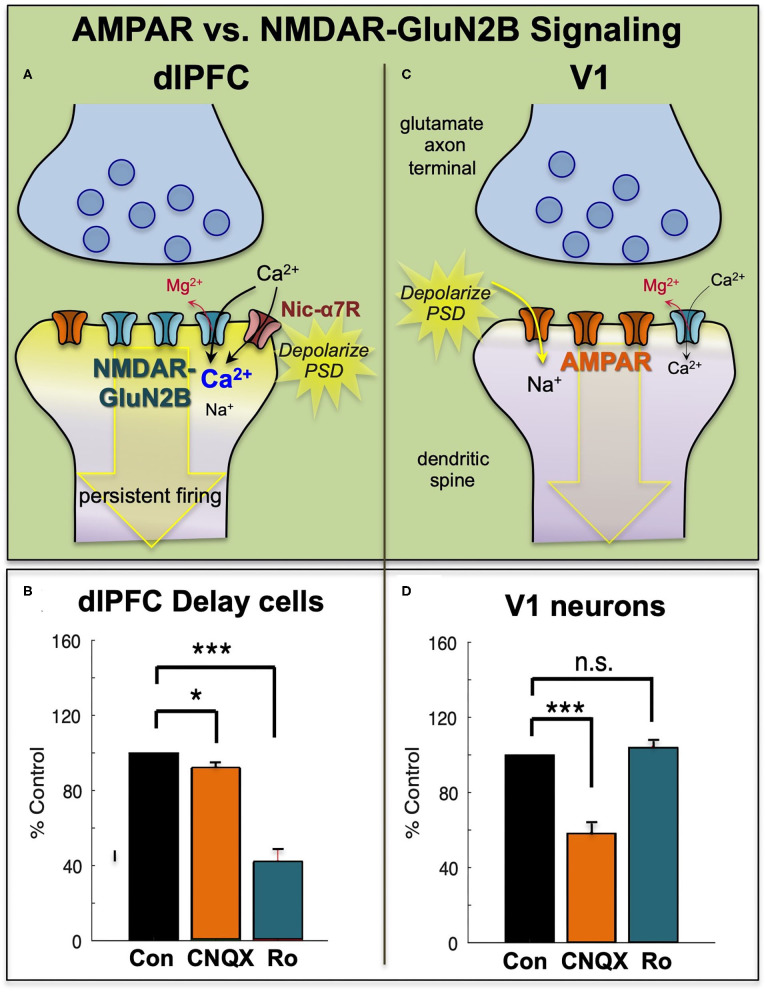
The primate dlPFC and primary visual cortex (V1) have very different neurotransmission. **(A)** The dlPFC depends on NMDAR neurotransmission, including those with slowly closing GluN2B subunits, that are exclusively within the PSD. The permissive excitatory effects to relieve the magnesium (Mg^2+^) block of the NMDAR ion channel are provided by acetylcholine (including Nic-a7R), with a surprisingly small influence from AMPAR. **(B)** Iontophoresis of the AMPAR antagonist, CNQX, has only subtle effects on dlPFC Delay cell firing, while blockade of NMDAR- GluN2B with Ro25-6981 (Ro) markedly reduces Delay cell firing. **(C)** Neurons in primate V1 show a more classic profile, relying heavily on AMPAR neurotransmission, with less influence by NMDAR. **(D)** Iontophoresis of low doses of the AMPAR antagonist, CNQX, markedly reduces V1 neuronal firing, while blockade of NMDAR- GluN2B with Ro has little effect. Adapted from (9) and (8). *p < 0.05, ***p < 0.001.

In contrast to NMDAR, blockade of AMPAR has remarkably subtle effects on Delay cell firing (7) (Figures 3A,B). This finding was initially confusing, as it is generally thought that AMPAR are essential to depolarize the PSD membrane and relieve the magnesium (Mg^2+^) block within the NMDAR pore, permitting NMDAR actions (Figure 3C). However, in dlPFC, this key permissive role appears to be played by acetylcholine acting at Nic-α7R and muscarinic M1R within the glutamate synapse (34, 35) which may depolarize the PSD to support persistent firing (Figures 3A,B). M1R may depolarize the PSD via closing of KCNQ channels localized in the PSD, and/or by enhancing levels of internal calcium release. These physiological data are consistent with behavioral data showing that Ach depletion from dlPFC is as deleterious as removing the cortex itself (36). As acetylcholine is released during wakefulness but not deep sleep, these mechanisms also help to coordinate cognitive state with arousal state, permitting conscious experience during wakefulness, but may render us unconscious during deep sleep when there is no acetylcholine release. Thus, as summarized in Figures 3A,B, Delay cell firing in dlPFC depends on NMDAR stimulation, including those with GluN2B subunits, with permissive actions by acetylcholine and more limited contributions from AMPAR.

AMPAR neurotransmission does play an important role in some dlPFC neurons that respond to sensory events, i.e., dlPFC Cue cells, and dlPFC response feedback cells that are thought to convey the corollary discharge back to dlPFC that the intended motor response has occurred (7). As these events require accurate timing, it is logical that they would have more of a reliance on rapid AMPAR neurotransmission.

Systemic ketamine treatment has differential effects on dlPFC neuronal firing depending upon their reliance on AMPAR vs. NMDAR neurotransmission. Consistent with their reliance on NMDAR neurotransmission, dlPFC Delay cells show *decreased* firing following systemic administration of the NMDAR antagonist, ketamine, at low doses used to treat intractable depression (7). This is only seen during cognitive performance and is not evident at rest. In contrast, systemic ketamine administration *increases* the spontaneous firing of response feedback neurons that rely on AMPAR (7), which resembles the increased firing seen with deep layer neurons in rat mPFC following NMDAR blockade, the basis for the “glutamate surge” (37). Some of this heterogeneity may arise from the balance of NMDAR on pyramidal cells vs. GABAergic interneurons, where pyramidal cell circuits with extensive recurrent NMDAR excitation may show loss of firing, while those circuits with extensive NMDAR on interneurons (e.g., in the primary sensory cortices) may have an overall increase in glutamate signaling. These data caution that ketamine's actions are heterogeneous, and that methods that average the response of large populations of neurons under resting conditions (e.g., resting fMRI, multi-electrode recording) may miss critical ketamine actions such as the loss of representations during working memory. The fact that ketamine's effects are circuit-specific creates a complicated picture, confounding our ability to identify the specific actions relevant to its antidepressant effects, distinguished from its actions that lead to cognitive disorder.

The importance of NMDAR transmission to the generation of mental representations needed for working memory and abstract thought may have relevance to a number of conditions where NMDAR are blocked or genetically weakened. The data from monkeys help to explain the profound cognitive alterations that can occur in the encephalitis arising from anti-NMDAR antibodies (38). The loss of mental representations with NMDAR blockade also helps to explain the profound cognitive impairments in schizophrenia where there can be genetic mutations that weaken NMDAR signaling (39), and/or blockade of NMDAR by kynurenic acid, especially under conditions of inflammation (40). Blockade of NMDAR by kynurenic acid may also contribute to cognitive deficits in Alzheimer's disease (41), given the importance of inflammatory signaling in early stages of disease. It is also possible that systemic infection may impair higher brain functions through the uptake of kyrurenine across the blood brain barrier (42). For example, the pervasive cognitive deficits in delirium might arise from high levels of kyrurenine crossing into the brain during systemic infection (43), and that the residual “brain fog” from infections such as COVID19 (44–46), which also leads to systemic kynurenine production, may also involve sustained blockade of NMDAR in higher cortical circuits by kynurenic acid. As there are pharmacological tools to reduce kynurenine production that may relieve NMDAR blockade, these are important areas for future research.

## NMDAR-GluN2B Expression Increases Across the Primate Cortical Hierarchy and Across Primate Evolution

There are multiple differences in function and physiology across the cortical hierarchy from primary sensory cortices, to association cortices to limbic cortices (Figure 1B). For example, there are increasing time scales in neuronal firing across the cortical hierarchy in rhesus monkeys (47) and in gray/white matter ratios in humans that correspond to transcriptional expression patterns (11). In particular, there is increasing expressing of the NMDAR GluN2B gene, *GRIN2B*, across the cortical hierarchy in humans, with low levels in primary visual cortex, high levels in dlPFC, and higher levels still in anterior cingulate cortices (11). As *GRIN2B* expression in dlPFC also increases across primate evolution, it suggests that this receptor plays an increasing role in primate mental experience.

Physiological studies in rodents (48) and monkeys are consistent with this hypothesis, as NMDAR-GluN2B has a much larger role in neurotransmission in the PFC than in the primary visual cortex, area V1. In rat medial PFC, the recurrent excitatory connections in layer V depend on NMDAR-GluN2B neurotransmission, while neurons in V1 showed much less reliance on these receptors (48). Similar results were seen in rhesus monkey dlPFC vs. V1. Neurons in V1 respond to the presentation of visual stimuli of a preferred orientation in their receptive field. These neurons have a great reliance on AMPAR transmission, where even low doses of AMPAR blockers such as CNQX markedly reduce stimulus-related firing (8) (Figures 3C,D). In contrast, high doses of NMDAR blockers are needed to reduce V1 neuronal firing [(8), Figures 3C,D]. A reliance on AMPAR stimulation is consistent with the function of V1 neurons, as the rapid kinetics of these receptors, in addition to their membrane properties (49), would allow accurate timing to encode the onset and offset of a sensory event. Thus, NMDAR transmission is not uniform across the primate cortex, and may be a feature of neurons requiring sustained neuronal firing for cognitive and possibly affective functions.

The very high levels of *GRIN2B* expression in the human anterior cingulate cortex (11) suggests that these receptor subtypes may be particularly important for the functioning of the cingulate cortices, e.g., in error detection, affective pain processing, and visceral affective responding. These limbic cortices and their corresponding connections are part of the neural networks that create “mood,” a sustained brain state. Given the role of NMDAR-GluN2B in mediating sustained firing in dlPFC, it is possible that these receptors have a parallel role in anterior and subgenual cingulate cortex. Although there are currently no direct iontophoretic recordings from primate anterior or subgenual cingulate cortex examining the role of GluN2B in cingulate physiology, this will be an important arena for future research. The following section outlines the importance of these receptors to cingulate processing of pain and visceral responding.

## The Role of NMDAR-GluN2B in the Cingulate Cortices Mediating Affective Pain Responses and Depression

The anterior cingulate (BA24) and subgenual cingulate (BA25) cortices mediate the emotional responses to pain [(14), reviewed in (2)], and are overactive in depression (50, 51). For example, the ACC is overactive in chronic pain and is a common ablation site for neurosurgical alleviation of intractable pain (52). In particular, BA25 in particular overactive in depression and a focus of deep brain stimulation (DBS) to relieve intractable depression (51). As described below, there is accumulating evidence that the emotional responses of the anterior and subgenual cingulate cortices rely on NMDAR-GluN2B neurotransmission, and that these aversive responses are reduced by ketamine administration in the treatment of chronic pain and depression.

Increasing evidence indicates that the response to pain in the rodent ACC (BA24) is mediated by NMDAR, including those with GluR2B subunits (53). GluR2B upregulate in response to chronic pain (54, 55), and long-term potentiation in the anterior cingulate cortex in response to painful stimuli is mediated by NMDAR-calcium-cAMP signaling, including NMDAR with GluR2B subunits, consistent with the sensitized response to chronic pain [reviewed in (56, 57)]. Systemic administration of ketamine, or of its active enatiomer, esketamine, reduces the response to pain as well as accompanying depressive symptoms in both rodents (58) and humans (59–62).

The subgenual cingulate (BA25) has extensive subcortical projections to mediate the emotional and visceral response to pain or other affective experiences (14), including to the lateral habenula (63), a nucleus activated by aversive events (64). Recent studies in marmosets have illuminated its functional role and relationship to ketamine treatment. These studies showed that pharmacological inactivation of BA25 decreased the autonomic and behavioral correlates of negative emotion expectation, while inactivation of BA32 increased them via generalization (27), consistent with BA32 providing top-down regulation of BA25. Conversely, activation of BA25 in marmosets induced an anhedonic state and reduced willingness to work for reward that was reversed by systemic administration of ketamine (65). 18F-FDG PET imaging of the marmosets showed that activation of BA25 was accompanied by activation of BA24 and insular cortex, while systemic ketamine treatment reduced the activation of these cortical areas (65). Over-activation of BA25 in marmosets also reduced vagal tone and heart rate variability, reduced the extinction of an aversive response and potentiated cortisol release during threat (66). Activation of BA25 in this study was associated with increased activity in the amygdala, the hypothalamus, and the temporal association area TH (66), but decreased the activity of the frontopolar cortex area 9, the dlPFC area 46, the central orbitofrontal cortex area13, and the lateral caudate (66). However, in this study, systemic ketamine did not reverse the effects of threat, suggesting that primitive responses to threat (e.g., in amygdala) may still control network activity. These data suggest that ketamine treatment may be most effective under conditions of safety. Research is still needed to determine how local infusion of ketamine into BA24 and/or BA25 alters emotional responding.

## Uncontrollable Stress Impairs Higher PFC Functions

**Figure 4:**
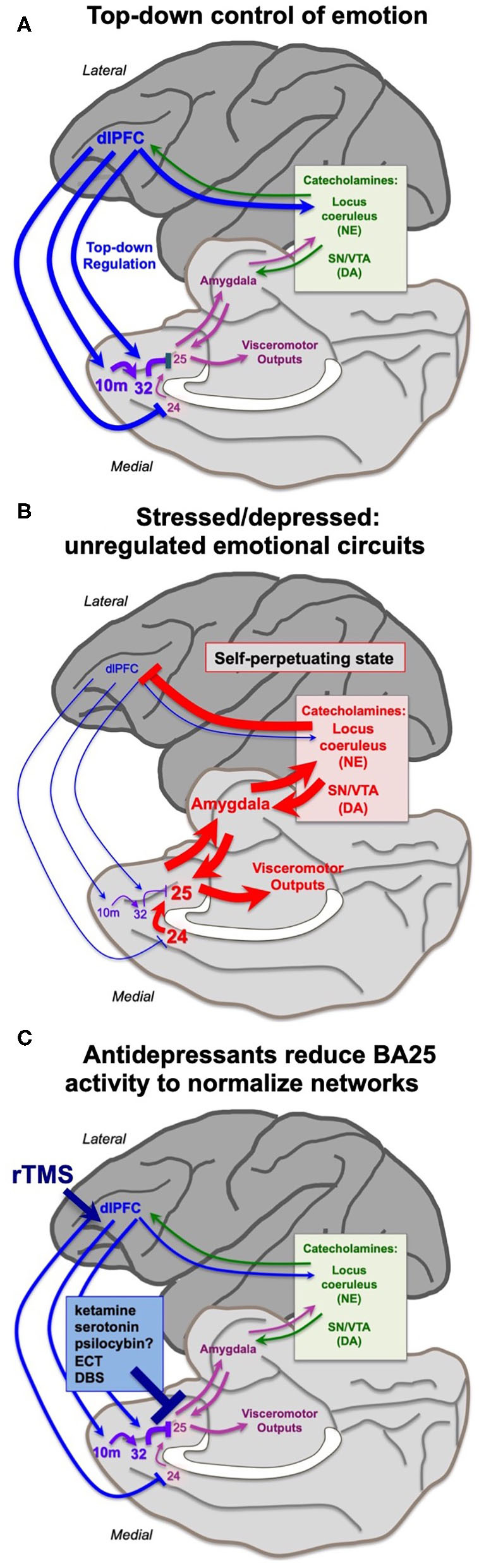
Hypothesis regarding the state of cortical circuits under conditions of health vs. depression, and their normalization by antidepressant treatments. (A) Under healthy conditions, the dlPFC and rostral medial PFC areas provide top-down regulation of the cingulate cortices via medial PFC connections, reducing BA25 activation of the stress response. The dlPFC also projects directly to the monoamine nuclei in the brainstem to regulate catecholamine release. **(B)** Under conditions of stress or depression, elevated activity in the cingulate cortices can activate the amygdala, and very high levels of catecholamine release in cortex takes higher PFC areas such as dlPFC “offline.” Thus, there is a self-perpetuating, unregulated state, where primitive circuits prevail. **(C)** Many antidepressant treatments reduce the activity of BA25. This may give the cortex a “foot in the door” to restore top-down regulation, especially when treatments promote dendritic spine restoration in higher PFC circuits. Other treatments may directly enhance the top-down regulation by the left dlPFC, e.g., rTMS and insight therapies.

The findings from the Roberts lab that activation of BA25 in marmoset reduces the activity of the rostral PFC and the dlPFC are consistent with a long line of research showing that these more newly evolved PFC areas are weakened by exposure to uncontrollable stress. As described above, under control conditions the dlPFC and rostral PFC can regulate emotion via projections to BA25 (Figures 1A, 4A), which in turn can control the activity of the brain's emotional circuits, including the amygdala, hypothalamus and brainstem (23, 25). A recent imaging study observed these rapid dynamics in human brain, where uncontrollable stress exposure initially reduced the activity of BA32, which then normalized in correspondence with reducing the stress response, and BA32 increased its functional connectivity with the dlPFC (67).

The more primitive cingulate and amygdala circuits may remove the top-down regulation by higher PFC circuits through activation of catecholamine neurons in the brainstem, which can weaken PFC connectivity. The PFC and cingulate cortices receive catecholamine innervation (68) and can also regulate the activity of the monoamine nuclei in the brainstem (18, 63, 69). The dlPFC requires moderate levels of catecholamines to function, but high levels of catecholamines released during even mild uncontrollable stress rapidly take the dlPFC “offline” [reviewed in (9, 70)]. Studies in rodents have shown that psychological stressors or threatening stimuli activate projections from the amygdala, e.g., to the locus coeruleus, increasing catecholamine release in the medial PFC (71–76). High levels of catecholamines in dlPFC drive feedforward calcium-cAMP signaling, opening nearby potassium (K^+^) channels on spines to rapidly weaken synaptic efficacy. This reduces the recurrent excitation underlying the persistent neuronal firing needed for mental representations [reviewed in (77, 78)]. High levels of glucocorticoids, released due to hypothalamic-pituitary-adrenal (HPA) actions, can also impair PFC working memory function (79), and may do so in part by blocking the extraneuronal catecholamine transporters on glia, which normally serve to reduce catecholamine levels in the extracellular space (80). In contrast to the dlPFC, high levels of catecholamines and glucocorticoids enhance the affective functioning of the amygdala (81–83), thus flipping the brain from a reflective to reflexive state. The rapid loss of dlPFC executive and working memory functions from a hypercatecholaminergic state has now been documented in humans (84–86) in addition to the original studies in rodents and monkeys (9, 77, 78). Thus, BA25 and amygdala can rapidly remove their regulation from higher order PFC circuits through activation of excessive catecholamine release in these higher PFC regions (Figure 4B). The cingulate cortices may also inhibit dlPFC by activating inhibitory GABAergic interneurons in the dlPFC (87).

This state of weakened higher PFC circuits and stronger BA24/BA25/amygdala control of brain responding is codified by chronic stress, which induces spine loss and dendritic retraction in PFC neurons which correlate with impaired working memory and attention regulation (88–90). Much of this research has been done in rats, where it is important to identify the projections of the neurons under study. Shansky's (91) elegant studies have shown that chronic stress exposure causes atrophy of cortico-cortical projecting mPFC neurons, but expands the dendrites of PFC neurons that activate the amygdala (i.e. those that are similar to primate BA25). Weaker connectivity and reduced gray matter in higher PFC circuits following chronic stress exposure has also been documented in humans (92, 93). Thus, chronic stress can create a self-perpetuating state where high levels of BA25/amygdala activity maintain a high catecholamine state, which simultaneously strengthens the amygdala but weakens higher PFC areas, removing inhibitory regulation of emotional response (Figure 4B). It is not known how catecholamines alter the activity of BA24 or BA25 in primates; this would be an important area for future research. Studies in rats have shown that the spine loss and dendritic retraction caused by chronic stress exposure can reverse with substantial time spent in a non-stressed state, at least in young animals, indicating a plastic dendritic response (94).

## Hypothesis: The Rapid Antidepressant Actions of Ketamine May Arise from Blockade of Mental Representations Generating Aversive Mood State in Cingulate Cortices

The loss of rostral PFC and dlPFC activity in concert with increased cingulate and amygdala activation would shift mental state from an outward, cognitively-engaged frame of mind to one focused inwardly on aversive experience. This is common in depression, where there is often loss of perspective, reduced empathy for others, anhedonia, and an urgent need for relief of mental anguish (95). Symptoms such as loss of motivation and psychomotor paralysis might also arise from BA25 activation of the peri-aqueductal gray and subthalamic nucleus that are positioned to reduce motor, cognitive and affective actions. Thus, the overactive subgenual cingulate must be inhibited to give more rostral PFC and dlPFC areas a “foot in the door” to regain regulation of the brain, including the regrowth of spines in higher PFC areas (96, 97), to restore top-down higher network connections.

We have hypothesized that ketamine interrupts the self-perpetuating cycle of primitive circuit activity that is sustained by BA25 overactivity, allowing higher PFC circuits the opportunity to restore more normal functioning [Figures 4B,C; (2)]. As noted by Mayberg (51), all effective antidepressant treatments, whether pharmacological (selective serotonin reuptake inhibitors (SSRIs), possibly psilocybin?), electrical (ECT, DBS) or cognitive (talk therapy, CBT), reduce BA25 hyperactivity in depressed patients (Figure 4C). rTMS (repetitive transcranial magnetic stimulation) to strengthen the functioning of the left dlPFC may also help to restore regulation of the cingulate cortices (Figure 4C), as the efficacy of this treatment correlates with reduced activity of the anterior cingulate cortex (98), and weaker connectivity of the subgenual cingulate cortex (99). The antidepressant effects of SSRIs may be related to the very high levels of serotonin transporters in BA25 (100), although research is still needed to determine the receptor mechanisms by which serotonin can inhibit BA25 neuronal firing. We have proposed that ketamine's therapeutic effects may arise from ultra-rapid inhibition of BA25 neurons (2). As described above, systemic ketamine administration can overcome the deleterious effects of BA25 over-activation in marmosets (65), and can also normalize BA25 hyperactivity in depressed subjects (101), which may involve blockade of NMDAR transmission in the cingulate circuits representing a sustained, aversive state. Ketamine also reduces burst firing in the habenula, which may also contribute to its ultrarapid therapeutic effects (64).

Intranasal ketamine or esketamine administration may produce ultra-rapid antidepressant effects by delivering the drug directly to the anterior and subgenual cingulate cortices, which reside directly caudal to the nasal epithelium (2). Ultra-rapid effects have been documented following this route of administration, with significant improvement at 40 min (102), maximal improvement at 24 h, with therapeutic effects waning, but still evident at 48 h post-administration (102). We have proposed that the initial improvement at 40 min would arise from NMDAR blockade of excessive neuronal firing in the anterior and subgenual cingulate cortices, allowing a restoration of regulation by higher PFC areas, where spine growth would provide more sustained antidepressant actions (2).

Support for this hypothesis comes from a remarkable recent rodent study, where dendritic spine changes in medial PFC could be monitored *in vivo* (103). Prolonged exposure to chronic unpredictable stress increased “depressive-like behaviors” in the mice, and caused a retraction of dendritic spines in the mPFC, while systemic administration of ketamine normalized behavior and restored spine density (103). However, this study found that ketamine improved behavior *prior* to spine re-emergence (103), suggesting that the initial beneficial effects may arise from alterations in neuronal firing, while the longer-term, sustained antidepressant response requires regrowth of spines in PFC circuits that provide top-down regulation. Finally, our data from the dlPFC in monkeys would suggest that ketamine levels would need to dissipate before full dlPFC function could be restored, given the reliance of layer III dlPFC circuits on NMDAR-GluN2B neurotransmission. This hypothesis would be consistent with the maximal therapeutic effects observed 24 h after ketamine administration.

In closing, we are learning that NMDAR transmission is especially important for persistent neuronal firing. It is possible that the sustained neuronal activity underlying mood state, and particularly an aversive mental state, similarly relies on NMDAR transmission, and thus is relieved by NMDAR blockade from ketamine.

## Author Contributions

All authors listed have made a substantial, direct and intellectual contribution to the work, and approved it for publication.

## Conflict of Interest

AA and Yale University receive royalties from Shire/Takeda from the USA sales of Intuniv. They do not receive royalties from generic or nonUSA sales. The remaining authors declare that the research was conducted in the absence of any commercial or financial relationships that could be construed as a potential conflict of interest.

## Abbreviations

ACC: anterior cingulate cortex
AMPAR: α-amino-3-hydroxy-5-methyl-4-isoxazolepropionic acid receptor, an ionotropic glutamate receptor
BA24: Brodmann's area 24, part of the anterior cingulate cortex
BA25: Brodmann's area 25, also known as the subgenual cingulate cortex
BA32: Brodmann's area 32, part of the ventromedial cortex
BA46: Brodmann's area 46, part of the dorsolateral prefrontal cortex
dlPFC: dorsolateral prefrontal cortex
GABA: Gamma-AminoButyric Acid, an inhibitory neurotransmitter
GluN2B: a subunit of the NMDAR, which closes slowly and fluxes high levels of calcium
HPA axis: Hypothalamus Pituitary Adrenal gland axis for control of cortisol release from the adrenal cortex (corticosterone in rodents)
immunoEM: Immunoelectron microscopy
PFC: prefrontal cortex
LIP: lateral intraparietal cortex specialized for analyzing visual space
M1R: cholinergic muscarinic M1 receptor
mPFC: medial prefrontal cortex
MT: middle temporal visual cortical area specialized for analyzing visual motion
Nic, α7R: cholinergic nicotinic α7 receptor
NMDAR: N-methyl-D-aspartate receptor, an ionotropic glutamate receptor
PFC: prefrontal cortex
PSD: postsynaptic density
V1: primary visual cortex.
